# Simultaneous posterior eyelid ptosis repair and strabismus surgery: a single-stage approach

**DOI:** 10.5935/0004-2749.20230020

**Published:** 2023

**Authors:** Mehmet Serhat Mangan, Ruveyde Bolac, Merve Beyza Yildiz, Serhat Imamoglu, Ece Turan Vural, Nursal Melda Yenerel

**Affiliations:** 1 Sadik Eratik Eye Institute, Haydarpasa Numune Education and Research Hospital, University of Health Sciences, Istanbul, Turkey

**Keywords:** Blepharoptosis/surgery, Amblyopia; Strabismus/surgery, Oculomotor muscles/surgery, Eyelids, Ophthalmologic surgical procedures/ methods, Blefaroptose/cirurgia, Ambliopia, Estrabismo/cirurgia, Músculos oculomotores/cirurgia, Pálpebras, Procedimentos cirúrgicos oftalmológicos/métodos

## Abstract

**Purpose:**

Blepharoptosis with coexisting strabismus can be observed in adults, and both these conditions affect cosmetic appearance and have psychosocial effects. Both also commonly require surgery, which is typically performed using a sequential approach. This study aimed to evaluate the efficacy of simultaneous Müller’s muscle-conjunctival resection with or without tarsectomy and strabismus surgery in adult patients with ptosis and coexisting strabismus.

**Methods:**

Patients with ptosis and coexisting strabismus who underwent simultaneous Müller’s muscle-conjunctival resection with or without tarsectomy and horizontal strabismus surgery were retrospectively evaluated. Analysis included measurement of the angle of deviation in prism diopters, margin reflex distance, eyelid height asymmetry, and complications following surgery. Success of Müller’s muscle-conjunctival resection with or without tarsectomy was defined as a margin reflex distance of ≥3.5 and ≤5 mm with a difference between the two upper eyelids of <1 mm. Strabismus success was defined as alignment with ±10 prism diopters of orthotropia.

**Results:**

The patients comprised three women and five men with a mean age of 37.12 years (range, 22-62 years). The strabismus stage of the surgery was performed first in all patients. Upper eyelid symmetry outcomes were assessed as perfect (<0.5 mm) in four patients and good (≥0.5 mm, <1 mm) in four patients. Success of Müller’s muscle-conjunctival resection with or without tarsectomy was achieved in six of eight patients (75%), and strabismus success was achieved in all patients. No revision eyelid or strabismus surgery was required following simultaneous surgery in any of the patients.

**Conclusion:**

Müller’s muscle-conjunctival resection with or without tarsectomy combined with strabismus surgery may be an alternative approach for use in patients with ptosis and coexisting strabismus.

## Abstract (pt)

**Objetivo:**

Blefaroptose e estrabismo podem ser coexistentes em adultos e ambos afetam a aparência estética e o domínio psicossocial. Ambos também geralmente requerem cirurgia, realizada tradicionalmente em uma abordagem sequencial. O objetivo do presente estudo foi avaliar a eficácia da execução simultânea da ressecção musculoconjuntival de Müller, com ou sem cirurgia de tarsectomia, e da cirurgia de estrabismo em pacientes adultos com ptose e estrabismo coexistentes.

**Métodos:**

Foram retrospectivamente avaliados pacientes com ptose e estrabismo coexistentes submetidos simultaneamente à ressecção musculoconjuntival de Müller, com ou sem tarsectomia, e à cirurgia de estrabismo horizontal. A análise incluiu a mensuração do ângulo de desvio das dioptrias de prisma, a distância do reflexo à margem, a assimetria da altura palpebral e quaisquer complicações após a cirurgia. A ressecção musculoconjuntival de Müller, com ou sem sucesso na tarsectomia, foi considerada bem-sucedida com uma distância reflexo-margem medindo entre 3,5 e 5 mm, e uma diferença entre as duas pálpebras superiores menor que 1 mm. O sucesso da cirurgia de estrabismo foi definido como um alinhamento com ± 10 dioptrias prismáticas de ortotropia.

**Resultados:**

Os pacientes foram 3 mulheres e 5 homens, com média de idade de 37,12 anos (faixa de 22 a 62 anos). A parte de estrabismo da cirurgia foi realizada primeiro em todos os pacientes. Os resultados da simetria palpebral superior foram avaliados como perfeitos (<0,5 mm) em 4 pacientes, bons (≥0,5 mm, <1 mm) em 4 pacientes e regulares (≥1 mm) em nenhum. A ressecção musculoconjuntival de Müller, com ou sem sucesso na tarsectomia, teve sucesso em 6 dos 8 pacientes (75%) e a intervenção para o estrabismo foi bem-sucedida em todos os pacientes. Não foi necessária cirurgia de revisão da pálpebra ou do estrabismo após a cirurgia simultânea em nenhum paciente.

**Conclusão:**

A ressecção musculoconjuntival de Müller, com ou sem tarsectomia, pode ser combinada com a cirurgia de estrabismo em uma abordagem alternativa para pacientes com ptose e estrabismo coexistentes.

## INTRODUCTION

Blepharoptosis sometimes coexists with strabismus in adults^(1)^. Strabismus and eyelid disorders can develop secondary to systemic diseases (such as Graves’ disease) or can be present since childhood. Patients with both of these conditions typically have limitations in their field of vision, with cosmetic appearance and psychosocial effects^(2,3,4,5,6)^. Surgical treatment is commonly required to correct both ptosis and strabismus. Although sequential surgery is conventional^(7)^, simultaneous surgery has recently been described as efficacious in the aesthetic rehabilitation of thyroid-associated orbitopathy^(8)^. Mc-Cracken et al.^(9)^ reported successful results with simultaneous eyelid retraction and strabismus surgery in adults both with and without thyroid-associated orbitopathy. Additionally, successful simultaneous correction of both congenital ptosis and strabismus has been reported in children^(10,11)^. However, no studies on simultaneous correction of ptosis and strabismus have been reported in adults without thyroid-associated orbitopathy.

Some patients prefer to undergo single-stage surgery and wish to receive general anesthesia. However, intraoperative eyelid adjustments can be difficult in patients undergoing simultaneous surgery under general anesthesia. The anterior approach (levator resection or frontalis suspension) is often preferred for ptosis repair in literature on the use of combined surgery in pediatric and adult patients^(8,9,10,11)^. An important consideration of the combined procedure performed under general anesthesia is the limited predictability of the postoperative eyelid position.

A preoperative phenylephrine test can be used to predict the final eyelid position, thereby eliminating the need for intraoperative eyelid adjustments achieved with Müller’s muscle-conjunctival resection (MMCR) with or without tarsectomy (MMCR±T). The aim of this study was to evaluate the efficacy of simultaneous MMCR±T and horizontal strabismus surgery in adult patients with ptosis and coexisting strabismus.

## METHODS

### Study design

This study was approved by the Institutional Review Board and was conducted in accordance with the tenets of the Declaration of Helsinki. Written informed consent was obtained from all patients. The patients consented to having an identifiable photograph published. Patients with ptosis and coexisting strabismus who underwent simultaneous MMCR±T and horizontal strabismus surgery between October 2016 and March 2021 were retrospectively reviewed using clinical charts.

The inclusion criterion for the study was ptosis with concurrent horizontal comitant strabismus (eso/exotropia). Patients meeting any of the following criteria were excluded: (1) vertical strabismus (hypo/hypertropia); (2) incomitant strabismus; (3) nystagmus; (4) levator function of <10 mm; (5) previous eyelid or strabismus surgery; or (6) conjunctival and/or ocular surface problems (e.g., ocular cicatricial pemphigoid or Stevens-Johnson syndrome).

### Patient evaluation

Complete ophthalmologic examinations, including eyelid measurements and ocular motility examinations, were performed on each patient. All clinical measurements were performed by the same examiner (M.S.M.). The angle of deviation of the prism diopters was measured using the alternate prism cover test. Levator function was determined from the amount of eyelid excursion from the downward gaze to the upward gaze while immobilizing the frontalis muscle. Margin reflex distance (MRD1)^(12)^ was measured to the nearest 0.5 mm with the patient in a sitting position, ensuring that the brow was stabilized to prevent recruitment of the frontalis muscle, using the same ruler and in the primary position of the gaze. MRD1 was measured with the patient fixating using the ipsilateral eye. The phenylephrine test consisted of the administration of two drops of 2.5% phenylephrine at 5-minute intervals into the upper conjunctival fornix of the eyelid to be operated (uni/bilateral). Five minutes after administration of the drops, the MRD1 was reevaluated. The amount of resection by MMCR±T was determined according to the phenylephrine test. According to the suggestion by Putterman and Urist^(13)^, the conjunctiva and Müller’s muscle were resected 8 mm in cases where the application of phenylephrine elevated the eyelid to the height of the other, nonptotic eyelid. If the phenylephrine test resulted in an undercorrection of 1 mm, 9 mm was resected, and if the test resulted in a 1 mm overcorrection, 7 mm was resected. If required, MMCR was combined with a tarsectomy to improve the elevation with the consideration that excision of 1 mm of the tarsus may cause a nearly 1 mm elevation of the eyelid^(14)^. If the patient had a negative response to phenylephrine, 9 mm of Müller’s muscle-conjunctiva and 2 mm of tarsus were excised.

The analysis included measurement of the angle of the deviation in the prism diopters, MRD1, and eyelid height asymmetry, in addition to the consideration of any complications following surgery. Postoperative measurements were conducted 2 months after surgery. Postoperative eyelid height asymmetry after surgery was evaluated as follows: the results were considered perfect when the difference between the two upper eyelids was <0.5 mm, a good outcome was when the difference between the upper eyelids was ≥0.5 but <1 mm, and a fair outcome was when the difference was equal to or more than 1 mm. The main outcome was surgical success for both the MMCR±T and strabismus surgery. MMCR±T success was defined as an MRD1 within ≥3.5 and ≤5 mm and a difference between the two upper eyelids of <1 mm. Strabismus success was defined as alignment within ±10 prism diopters of orthotropia.

### Surgical procedures

All surgeries were performed by a single surgeon (M.S.M.), with the patient under general anesthesia. The strabismus stage of the surgery was performed first for all patients. The strabismus surgery consisted of a medial and/or lateral rectus recession and/or resection performed with a limbal-based incision unilaterally or bilaterally based on the type of strabismus (eso/exotropia) and angle of deviation (Table 1). The limbal-based incision was tightly closed with a 6-0 polyglactin suture. No bare sclera was left to minimize the risk of adhesion formation. The adjustable suture technique was not used.

**Table 1:** Clinical features of patients with blepharoptosis with coexisting strabismus who underwent simultaneous Müller’s muscle-conjunctival resection with or without tarsectomy and horizontal strabismus surgery

Case no	Sex	Age, years	F/U, m	LF, mm	MRD1 before surgery, mm	MMCR±T, side of the surgery	MMCR±T, amount of resection, mm	MRD1 after surgery, mm	Deviation, PD, before surgery	Strabismus surgery, side, amount, type	Deviation, PD, after surgery
1	F	22	11	16, 16	2.0, 4.0	OD	8	4.0, 4.0	50 XT	OD, 8-mm, LRRec & OD, 6-mm, MRRes	Ortho
2	F	32	6	13, 13	4.5, 3.0	OS	8	4.5, 4.5	60 XT	OS, 8-mm, LRRec & OS, 7-mm, MRRes	Ortho
3	M	29	7	17, 17	4.0, 1.5	OS	9	3.5, 3.0	25 ET	OS, 4-mm, MRRec & OS, 6-mm, LRRes	Ortho
4	M	34	10	11, 16	0.5, 4.0	OD	9 ± 1	3.0, 3.5	30 XT	OD, 6-mm, LRRec & OS, 6-mm, LRRec	5 XT
5	F	30	6	15, 15	4.5, 1.0	OS	9	4.0, 3.5	35 ET	OS, 5-mm, MRRec & OS, 6-mm, LRRes	5 ET
6	M	37	8	12, 16	1.5, 4.0	OD	8	3.5, 3.5	25 XT	OD, 5-mm, LRRec & OS, 5-mm, LRRec	Ortho
7	M	51	8	14, 14	2.0, 2.0	OU	9, 9	4.0, 4.0	45 ET	OS, 6-mm, MRRec & OS, 8-mm, LRRes	5 ET
8	M	62	6	11, 13	-0.5, 0	OU	9 ± 2, 9 ± 2	2.5, 3.0	55 XT	OD, 8-mm, LRRec & OD, 6-mm, MRRes	5 XT

M= male; F= female; F/U= follow-up; m= month; LF= levator function; MRD1= margin reflex distance; OD= right eye; OS= left eye; OU= both eye; MMCR±T= Müller’s muscle-conjunctival resection with or without tarsectomy; PD= prism diopters; ET= esotropia; XT= exotropia; Ortho= orthotropia; MRRec= medial rectus recession; MRRes= medial rectus resection; LRRec= lateral rectus recession; LRRes= lateral rectus resection.

For the MMCR±T, the eyelid was everted by a Desmarres retractor. After the desired amount of resection was marked and grasped with a Putterman ptosis clamp, it was secured with a 6-0 polypropylene suture in a continuous horizontal mattress pattern. The conjunctival and Müller’s muscle ± tarsus were excised, and the suture was externalized and tied over a bolster. Excision of the upper eyelid skin was not performed for any patient. A bandage contact lens was applied to the cornea at the end of the surgery. The suture was removed on the 10^(th^ day after surgery.

## RESULTS

Clinical features of the patients are presented in table 1. The patients comprised three women and five men with a mean age of 37.12 years (range, 22-62 years). The mean preoperative levator function was 13.6 and 15.5 mm for the operated and nonoperated eyes, respectively. Two patients had involutional ptosis, and six patients had congenital ptosis. Five patients had exotropia and three had esotropia before the surgery. Three patients were excluded from the study for vertical strabismus, two were excluded for incomitant strabismus, one for nystagmus, three for levator function of <10 mm, two for previous eyelid or strabismus surgery, and two for conjunctival and/or ocular surface problems.

**Figure 1:**
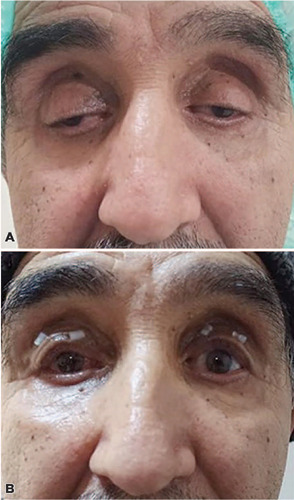
(A) Preoperative appearance of a 62-year-old man with bilateral severe blepharoptosis with coexisting right exotropia. (B) View of the patient after simultaneous bilateral Müller’s muscle-conjunctival resection with tarsectomy and right horizontal strabismus surgery.

**Figure 2:**
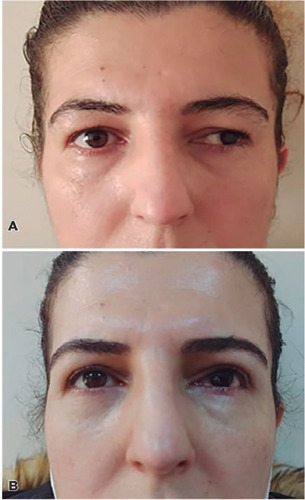
(A) Preoperative appearance of a 32-year-old woman with left mild blepharoptosis with coexisting left exotropia. (B) View of the patient after simultaneous left Müller’s muscle-conjunctival resection and left horizontal strabismus surgery.

In total, eight patients who underwent simultaneous MMCR±T and strabismus surgery were analyzed in this study (Figures 1A, 1B, 2A, and 2B). A tarsectomy was also performed in two patients. The mean preoperative MRD1 was 1.3 mm (range, -0.5 to 3 mm) and 4.1 mm (range, 4-4.5 mm) in the operated and nonoperated eyes, respectively. The mean postoperative MRD1 was 3.5 mm (range, 2.5-4.5 mm) and 3.8 mm (range, 3.5-4.5 mm) in the operated and nonoperated eyes, respectively. Symmetry outcomes were assessed as perfect (<0.5 mm) in four patients, good (≥0.5 mm, <1 mm) in four, and fair (≥1 mm) in none. Successful MMCR±T was achieved in six of the eight patients (75%), and successful strabismus was achieved in all. No revision eyelid or strabismus surgery was required following simultaneous surgery in any of the patients.

One case had minimal transient lagophthalmos after surgery. During the postoperative follow-up, no obvious corneal epitheliopathy, keratitis, or adhesions between the palpebral and bulbar conjunctiva were observed in any of the patients. The bandage contact lens was well tolerated in all patients.

## DISCUSSION

Adult strabismus is present in 4% of the population^(2)^. The main purposes of adult strabismus surgery are to achieve favorable cosmetic alignment of the eyes and rehabilitation or improvement of binocular vision. In addition to an improved cosmetic appearance, several studies have indicated significant benefits to patients’ psychosocial function through improvement in interpersonal relations, employment, exercise, and self-image^(2,3,4,5,6)^. The causes of strabismus in the adult population are numerous^(2)^; however, all patients in our study had untreated childhood horizontal strabismus.

Horizontal strabismus is more common than vertical strabismus in the general population^(2)^. A controversial issue regarding strabismus surgery is that patients undergoing this procedure may experience changes in eyelid position. Reports have demonstrated that eyelid position alterations following strabismus surgery are minimal (1-2 mm changes) and only involved the lower eyelid^(15,16)^.

In contrast, McCracken et al.^(9)^ reported that simultaneous eyelid and strabismus surgery did not diminish the results of either procedure. They noted that eyelid surgery can be performed simultaneously with horizontal strabismus surgery, but they did not recommend a combination with vertical strabismus surgery^((9)^. Bernardini et al.^((8)^ recently reported that secondary eyelid operations were not required after strabismus surgery, confirming that the eyelid position was not affected by the surgery. In our study, all patients had horizontal strabismus, and no revision eyelid surgery was performed following simultaneous surgery. The strabismus stage of the surgery was performed first, and a lid speculum was not used following eyelid surgery. This approach may have led to improved outcomes. Revere et al.^(11)^ stated that the use of a lid speculum after eyelid surgery might negatively affect the eyelid position due to stretching of the sutured incision line.

Despite staged surgery being the conventional approach, no evidence in the literature demonstrates any advantage of performing it in patients with strabismus and ptosis^(8,9,10,11)^. Staged surgery has disadvantages, including that the patient must undergo multiple surgeries and anesthesia, as well as requiring increased cost and effort. Bernardini et al.^(8)^ recently described a novel approach for aesthetic rehabilitation of thyroid-associated orbitopathy and reported that simultaneous surgery yielded successful results and high patient satisfaction. Likewise, McCracken et al.^((9)^ reported that simultaneous eyelid retraction and strabismus surgery was performed successfully in nine adult patients with or without thyroid-associated orbitopathy. Similar to the results in adults, previous studies have documented satisfactory clinical results following simultaneous ptosis and strabismus surgery in children^(10,11)^. Zhou et al.^(10)^ described 10 children with congenital ptosis and strabismus who underwent external levator resection or frontalis suspension. Another study by Revere et al.^(11)^ reported a success rate of 75% with simple congenital ptosis following frontalis suspension, external levator resection, or modified Fasanella-Servat surgery. In this study, simultaneous surgery was performed in adult patients without thyroid-associated orbitopathy or vertical strabismus. MMCR±T success was achieved in 75% of the patients, and strabismus surgery was successful in all. No revision eyelid or strabismus surgery was required in any patient following simultaneous surgery, and all cases had good or perfect upper eyelid height symmetry afterward.

The present study group consisted of patients who wished to have single-stage surgery under general anesthesia for several reasons. Importantly, simultaneous surgery under general anesthesia can be difficult with respect to intraoperative eyelid adjustments. Additionally, it is important to determine which eyelid correction approach is more effective for patients with ptosis and coexisting strabismus. The primary problem in these cases is achieving the desired postoperative eyelid height symmetry and limited predictability of the eyelid position after surgery; patients undergoing levator resection can experience a change in eyelid position, and this is not entirely predictable^(17)^. In our study, none of the patients had an overcorrection. The achievement of a satisfactory result in the current study may be a consequence of using the preoperative phenylephrine test to predict the final contralateral eyelid position. This eliminated the need for intraoperative eyelid adjustments, whereas the amount of tissue to resect was determined by the amount of ptosis correction after a comparison of both eyelid heights. Although MRD1 varied in the phenylephrine test before surgery, our findings may support the recommendation to use a previously described algorithm and technique^(13,14)^ to achieve optimal upper eyelid height and eyelid height symmetry after surgery in most patients. In our study, one patient had a negative response to phenylephrine before surgery. This patient underwent a bilateral 9-mm Müller’s muscle-conjunctiva and a 2-mm tarsus excision and achieved a satisfactory result. Previous studies have reported that MMCR may have a broader application for ptosis patients with a negative response to phenylephrine^(18,19)^. Furthermore, the addition of a tarsectomy to the MMCR procedure improved the upper eyelid height position^(14,20,21)^. Nemet^((22)^ also reported that the Hering’s law effect is more common with the levator approach than with MMCR. Consistent with that study^(22)^, MMCR±T may be more predictable than levator resection surgery.

McCracken et al.^(9)^ did not recommend transconjunctival eyelid surgery because there is the possibility of adhesions between the palpebral and bulbar conjunctiva; however, they did not perform transconjunctival eyelid surgery in their series. In contrast, Revere et al.^(11)^ reported no adhesion formation in any patients who underwent modified Fasanella-Servat surgery or in a subgroup analysis of isolated superior conjunctival incisions. In addition, another study indicated that simultaneous surgery of the vertical rectus muscle (superior conjunctival incisions) and adjacent eyelid did carry a risk of adhesion formation^(9)^. A case report by Simpson et al.^(23)^ described simultaneous levator muscle recession by a posterior approach and lateral rectus muscle recession in an adult patient with thyroid-associated orbitopathy and upper eyelid retraction. Adhesion was observed between the tarsal conjunctiva and the globe; however, they left a bare area of the sclera during the surgery, and this may predispose to adhesion formation. In our study, no adhesion formation was observed in any patient, which may reflect the close suturing of the conjunctiva and the fact that all the included cases had horizontal strabismus without thyroid-associated orbitopathy. Contact among the cornea, eyelid, and conjunctiva sutures may increase epitheliopathy and inflammation with eye blinking. Hence, we used a bandage contact lens in all patients to eliminate the mechanical effects of the upper eyelid on the globe and protect the cornea, limbus, and adjacent bulbar conjunctiva. Furthermore, patients with thyroid-associated orbitopathy may have inflammation of the conjunctiva and periocular tissue, which may make them prone to adhesion formation following simultaneous eyelid and strabismus surgery.

The limitations of our study include small number of patients; nonrandomized, retrospective design; and absence of a comparative group. Clinically, measurement of the MRD1 may have reduced objective evaluation, and thus there may have been an increased risk of bias.

To our knowledge, this article is the first study to report simultaneous MMCR±T and horizontal strabismus surgery in patients with ptosis and coexisting strabismus. Use of the phenylephrine test to predict the final eyelid position, together with a previously described algorithm and technique^(13,14)^ for MMCR±T, resulted in good surgical outcomes for most patients. MMCR±T is easy to perform and safe, can be completed quickly, eliminates the need for patient cooperation, and protects the anterior lamella, thereby reducing the risk of eyelid sensation^((24)^. Therefore, MMCR±T combined with strabismus surgery may represent an alternative approach for use in patients with ptosis and coexisting strabismus. However, this approach must be supported by prospective and comparative studies on larger patient series.
